# Impact of Minimal Dose Strategy Before Antithyroid Drug Discontinuation on Relapse Risk in Graves’ Disease

**DOI:** 10.1210/clinem/dgaf433

**Published:** 2025-08-01

**Authors:** Keitaro Miyamura, Mitsuru Ito, Hiroyuki Yamaoka, Mako Hisakado, Eijun Nishihara, Shuji Fukata, Mitsushige Nishikawa, Akira Miyauchi, Takashi Akamizu

**Affiliations:** Center for Excellence in Thyroid Care, Kuma Hospital, Kobe 650-0011, Japan; Center for Excellence in Thyroid Care, Kuma Hospital, Kobe 650-0011, Japan; Center for Excellence in Thyroid Care, Kuma Hospital, Kobe 650-0011, Japan; Center for Excellence in Thyroid Care, Kuma Hospital, Kobe 650-0011, Japan; Center for Excellence in Thyroid Care, Kuma Hospital, Kobe 650-0011, Japan; Center for Excellence in Thyroid Care, Kuma Hospital, Kobe 650-0011, Japan; Center for Excellence in Thyroid Care, Kuma Hospital, Kobe 650-0011, Japan; Center for Excellence in Thyroid Care, Kuma Hospital, Kobe 650-0011, Japan; Center for Excellence in Thyroid Care, Kuma Hospital, Kobe 650-0011, Japan

**Keywords:** Graves’ disease, antithyroid drugs, methimazole, relapse risk, maintenance dose, retrospective cohort study

## Abstract

**Background:**

Although antithyroid drugs (ATDs) are widely used as the first-line therapy for Graves’ disease, relapse after treatment discontinuation remains common. While a prolonged low-dose ATD therapy has been associated with improved remission rates, the impact of the minimal maintenance dose before discontinuation on relapse risk remains unclear.

**Method:**

We conducted a retrospective cohort study using electronic medical records from a thyroid specialty hospital in Japan. Patients newly diagnosed with Graves’ disease between 2008 and 2024 who had discontinued methimazole (MMI) after receiving a minimal maintenance dose (≤2.5 mg/day) were included. Patients were categorized into 4 groups based on their final maintenance dose before discontinuation: 2.5 mg/day, >1.25 to ≤2.5 mg/day, 1.25 mg/day, and <1.25 mg/day. We evaluated the association between the minimal MMI dose before discontinuation and the 1-year risk of relapse using multivariable regression and propensity score–matched analyses.

**Results:**

Among 4352 eligible patients, multivariable regression showed that, compared with the 2.5 mg/day group, the 1.25 mg/day group had a significantly lower risk of relapse within 1 year [risk ratio (RR) 0.46, 95% confidence interval (CI) 0.28-0.75], and the <1.25 mg/day group had the lowest risk (RR: 0.18, 95% CI: 0.05-0.73). The propensity score-matched analysis, consistent with the multivariable regression, showed that the 1.25 mg/day group had a lower risk of relapse compared with the 2.5 mg/day group (RR: 0.44, 95% CI: 0.23-0.85; 172 matched pairs).

**Conclusion:**

Lower minimum maintenance doses of MMI before discontinuation, particularly doses <2.5 mg/day, may be associated with a reduced risk of relapse in patients with Graves’ disease. Clinicians should recognize the clinical relevance of the minimum maintenance dose in the treatment of Graves’ disease.

Graves’ disease is the most common cause of hyperthyroidism, with an incidence of approximately 20 to 50 per 100 000 person-years in the general population, varying by region and iodine intake (1), and a lifetime cumulative risk of 3.8% in women and 0.9% in men (2). The treatment of Graves’ disease has globally relied on antithyroid drugs (ATDs) as first-line therapy (3, 4). This preference is supported by increasing reports on the long-term safety of ATDs (5) and the improvement in remission rates associated with prolonged administration of ATDs (6).

Achieving and maintaining remission remains one of the major challenges in the management of Graves’ disease. Despite the widespread use of ATDs, relapse after ATD withdrawal remains frequent, with rates ranging from 20% to 70%, depending on the population and study conditions (4, 7, 8). Given these outcomes, optimizing treatment strategies to reduce the risk of relapse in Graves’ disease is critical, especially in identifying the most effective dosing regimen before drug discontinuation.

Several factors influencing the risk of relapse have been identified, including thyrotropin receptor antibodies (TRAb) seroconversion (9), total ATD treatment duration (6), and the length of time patients remain on a minimal dose (10). Recent studies suggest that prolonged treatment with low-dose ATDs is associated with cumulative improvements in remission rates (6). As more patients receive long-term ATD therapy, the minimum maintenance dose before discontinuation emerges as a critical, modifiable factor under the control of physicians. However, despite its apparent importance, a significant gap in the literature remains regarding the evaluation of a minimal maintenance treatment dose before discontinuing ATD therapy.

The minimal maintenance dose of methimazole (MMI) before discontinuation has been easier to taper in Japan since a 2.5 mg tablet formulation in February 2021 became available. This recent development enables direct investigation into whether reducing the maintenance dose below the conventional 2.5 mg/day (11) can mitigate the risk of relapse. Clarifying whether further dose reduction before discontinuation can meaningfully reduce relapse risk is essential for informing evidence-based strategies to prevent relapse. Our thyroid specialty hospital, which manages many patients with Graves’ disease and accumulates high-quality, real-world data through routine clinical care, provides an ideal setting to investigate how lower maintenance doses might influence relapse risk.

This retrospective study aimed to evaluate whether a lower maintenance dose of MMI before discontinuation is associated with a reduced risk of relapse in patients with Graves’ disease.

## Materials and Methods

### Study Design and Participants

This retrospective cohort study was conducted using electronic medical records of patients newly diagnosed with Graves’ disease at our institution between August 1, 2008, and July 31, 2024, after we transitioned to the electrochemiluminescence immunoassay method for measuring TRAb. This study followed the Strengthening the Reporting of Observational Studies in Epidemiology statement (12). The study was conducted at a single-center community hospital specializing in thyroid diseases in Hyogo Prefecture, Japan. The hospital treats approximately 5000 patients with Graves’ disease monthly, including about 125 new cases. Patients are referred not only from Hyogo Prefecture but also from surrounding prefectures, indicating that the study population included a broad range of cases representative of clinical practice in Japan. We identified patients with International Classification of Diseases, Tenth Revision code E050 and extracted those who had “Graves’ disease” registered as the diagnosis in the hospital's Diagnosis Procedure Combination system, which is a case-mix classification system based on the International Classification of Diseases, Tenth Revision in Japan (13). From this population, we selected patients who had discontinued MMI after receiving at least 2 consecutive prescriptions of MMI at a stable maintenance dose of 2.5 mg/day or lower. Patients were excluded if they discontinued MMI following pregnancy, thyroidectomy, or radioiodine therapy. We also excluded patients who maintained a minimal MMI dose for <3 months, as this deviates from Japanese guidelines recommending at least 6 months (11, 14). Furthermore, patients whose last visit was within 6 months after MMI discontinuation were excluded due to a high likelihood of loss to follow-up.

### Discontinuation of MMI

To validate whether the MMI discontinuation cases extracted for this study truly reflected stable control of Graves’ disease, a validation study was conducted. MMI discontinuation was confirmed based on both normal thyroid function or subclinical hypothyroidism at the time of cessation and explicit documentation of “discontinuation” or “drug withdrawal” in the medical records.

To ensure adequate precision in evaluating the accuracy of our case definition for treatment discontinuation, we randomly sampled 150 cases. This sample size was determined to include at least 100 true positive cases based on an expected positive predictive value of approximately 90% and to achieve a 95% confidence interval (CI) with a margin of error of around ±10% (15). A review of electronic medical records confirmed that 139 of these cases represented true discontinuation due to stable disease control, yielding a positive predictive value of 92.6% (139/150), indicating satisfactory reliability. The 11 misclassified cases mainly reflected database-related limitations, such as leftover medication, external prescriptions, and misattribution of non-ndocrine visits as discontinuation events.

### Relapse of Graves’ Disease

Relapse of Graves’ disease was defined as the resumption of ATD therapy within 1 year after discontinuation. While the American Thyroid Association guideline defines remission as maintaining normal thyroid function for 1 year after discontinuation (8), cases of transient thyrotoxicosis following withdrawal of ATDs have also been reported (16). Furthermore, it was not feasible in this study to directly monitor thyroid function longitudinally over the 1-year period following discontinuation. Therefore, resuming ATD therapy was used as a surrogate indicator of relapse.

### Covariates

Based on previous research and a directed acyclic graph (17) [Supplementary Figure S (18)], we selected the following covariates for calculating the propensity score: age at diagnosis of Graves’ disease, sex, smoking history, thyroid volume, duration of minimal maintenance dose of MMI, total treatment duration from MMI initiation to discontinuation, and TRAb titer at the time of MMI discontinuation. Age at starting MMI was categorized as <18, 18 to 40, and ≥40 years, considering reports of higher relapse rates in pediatric patients (19) and those <40 years (20-22). The risk of relapse is reportedly higher in males (20-22). Smoking status at the time of initial visit was classified into “Yes” and “No” groups, as smoking has been associated with a higher risk of relapse (23). Thyroid volume was classified into 3 groups (0-20 g, 20-80 g, and ≥80 g) based on previous reports suggesting that a larger thyroid volume may be associated with an increased risk of relapse (8, 20, 23). Volume was estimated by ultrasound using the ellipsoid formula: π/6 × length × width × depth. This formula was applied uniformly, although the estimate may be affected in cases with large or protruding thyroid nodules. The minimal maintenance dose duration of MMI was categorized into 3 groups (3 months to <6 months, 6 months to <1 year, ≥1 year), considering its distribution in the study population and Japanese treatment guidelines recommending a minimum of 6 months (11). Since longer treatment duration has been associated with an increased cumulative remission rate (6), treatment duration was treated as a continuous variable. TRAb titers at the time of MMI discontinuation were categorized as < detection limit or ≥ detection limit, as nonnegative TRAb levels have been associated with a higher risk of relapse (9). A “missing” category was assigned to each variable with missing data for inclusion in the analysis. Initial free T4 and TRAb levels were not included as covariates, as they were not considered confounders due to the lack of clear association with the minimal maintenance dose of MMI.

### Statistical Analyses

We analyzed the association between the maintenance dose of MMI before discontinuation and the 1-year risk of relapse in patients with Graves’ disease using multivariable regression analysis. MMI dose was categorized into 4 groups: ≤1.25 mg/day, exactly 1.25 mg/day, >1.25 to <2.5 mg/day, and 2.5 mg/day, with the 2.5 mg/day group designated as the reference category. For patients receiving nondaily dosing regimens, the average daily dose was calculated by dividing the total weekly dose by 7. For example, a regimen of 5 mg taken twice per week was estimated as (5 mg × 2) ÷ 7 ≈ 1.43 mg/day, and patients were categorized accordingly. Since the risk of relapse was 13.1%, we used modified Poisson regression with robust variance estimation to calculate risk ratios (RRs) for each dose category (24).

### Sensitivity Analysis

To assess the robustness of the primary findings, we conducted sensitivity analyses using alternative analytic strategies. First, we performed propensity score matching using the same exposure definition and covariates as in the primary analysis. The propensity score was calculated with a caliper of 0.01, and 1:1 matching was performed. We initially estimated the risk of relapse for the MMI 1.25 mg/day group, using the MMI 2.5 mg/day group as the reference. To assess the potential impact of unmeasured confounding, we calculated the E-value, which quantifies the minimum strength of association that an unmeasured confounder would need to have with both the exposure (minimal maintenance dose of MMI) and the outcome (relapse of Graves’ disease) to fully explain away the observed association. The E-value was computed based on the RR and its CI limits (25). Next, we estimated the RR for the group receiving ≤1.25 mg/day, using the >1.25 to ≤2.5 mg/day group as the reference and applying the same propensity score–matching approach.

As an additional sensitivity analysis, we repeated the propensity score-matched analyses in a restricted cohort of patients whose interval from MMI discontinuation to their last visit was ≥1 year, ensuring sufficient observation time to minimize potential misclassification of relapse-free status. We also excluded 108 patients who received levothyroxine at the time of MMI discontinuation and repeated the same multivariable analysis in the remaining 4244 patients to assess the impact of cotreatment.

Data analyses were carried out using STATA version 15 (Stata Corp LP, College Station, TX, USA).

### Ethical Approval and Informed Consent

This study was approved by the Ethics Committee of Kuma Hospital (No. 20200709-1), and the need for informed consent was waived based on the retrospective study design.

## Results

**Figure 1.**
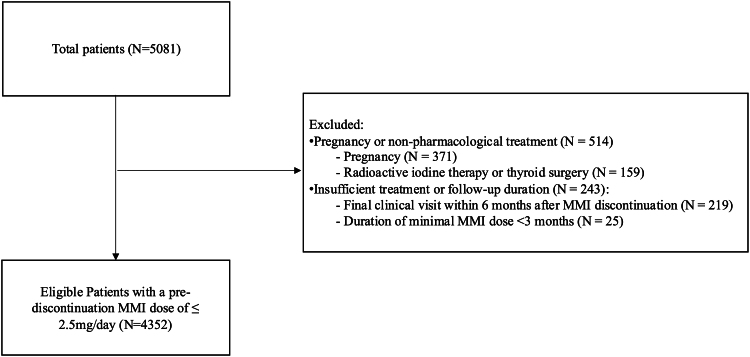
Flow chart of patient selection. Abbreviation: MMI, methimazole.

From August 2008 to July 2024, a total of 5081 patients who were initiated on MMI for the management of Graves’ disease and subsequently discontinued MMI treatment were identified. Among these, 371 patients with a history of pregnancy before MMI discontinuation and 159 patients who underwent radioactive iodine therapy or thyroidectomy before discontinuation were excluded (Fig. 1). Regarding follow-up after discontinuation, 219 patients who had been followed up for less than 1 year after MMI withdrawal, and 25 patients who had maintained the minimum dose of MMI for less than 3 months before discontinuation were also excluded. The remaining 4352 patients, all of whom had been maintained on MMI doses of <2.5 mg/day before discontinuation, were included in the analysis. Among them, 3750 patients had received either 1.25 mg/day or 2.5 mg/day before discontinuation.

Baseline characteristics of patients stratified by prediscontinuation MMI dose are presented in Table 1. The total cohort included 4352 patients divided into 4 groups: <1.25 mg/day (n = 76), 1.25 mg/day (n = 227), >1.25 to <2.5 mg/day (n = 526), and 2.5 mg/day (n = 3523). The 1-year relapse rates were 2.6%, 7.1%, 13.1%, and 13.8% across these groups, respectively, showing an inverse trend between MMI dose and relapse risk. The duration of MMI treatment tended to be longer in the lower-dose groups. Other baseline variables, including age, sex, smoking status, and thyroid volume, were generally comparable across groups. TRAb titers below the detection limit were more frequently observed in the lower-dose groups.

**Table 1.** Baseline characteristics

	Total	Prediscontinuation MMI dose^*a*^			
		< 1.25 mg/day	1.25 mg/day	>1.25 to <2.5 mg/day	2.5 mg/day
	n	n (%)	n (%)	n (%)	n (%)
Total	4352	76	227	526	3523
1-year relapse of Graves’ disease					
Yes	572	2 (2.6)	16 (7.1)	69 (13.1)	485 (13.8)
No	3780	74 (97.4)	211 (93.0)	457 (86.9)	3038 (86.2)
Sex					
Male	789	17 (22.4)	44 (19.4)	90 (17.1)	638 (18.1)
Female	3563	59 (77.6)	183 (80.6)	436 (82.9)	2885 (81.9)
Age at treatment initiation					
<18 years	140	0 (0.0)	6 (2.6)	30 (5.7)	104 (3.0)
18-40 years	1277	17 (22.4)	54 (23.8)	130 (24.7)	1076 (30.5)
≥40 years	2935	59 (77.6)	167 (73.6)	366 (69.6)	2343 (66.5)
Thyroid volume					
< 20 g	1440	28 (36.8)	83 (36.6)	189 (35.9)	1140 (32.4)
≥ 20 g to < 80 g	2623	45 (39.2)	130 (57.3)	304 (57.8)	2144 (60.9)
≥ 80 g	78	1 (1.3)	2 (0.9)	12 (2.3)	63 (1.8)
Missing	211	2 (2.6)	12 (5.3)	21 (4.0)	176 (5.0)
Smoking					
Yes	714	10 (13.2)	35 (15.4)	84 (16.0)	585 (16.6)
No	3585	66 (86.8)	189 (83.3)	432 (82.1)	2898 (82.3)
Missing	53	0 (0.0)	3 (1.3)	10 (1.9)	40 (1.1)
Duration of MMI treatment (years)*	2.7	4.8 (2.9)	3.8 (2.6)	3.4 (2.1)	2.5 (1.9)
Duration of minimal MMI dose					
≥ 3 months to <6 months	793	17 (22.4)	41 (18.1)	120 (22.8)	615 (17.5)
≥ 6 months to < 1 year	1864	40 (52.6)	149 (65.6)	240 (45.6)	1435 (40.7)
≥ 1 year	1695	19 (25.0)	37 (16.3)	166 (31.6)	1473 (41.8)
TRAb titer at the time of MMI discontinuation					
< detection limit	2135	43 (56.6)	194 (85.5)	285 (54.2)	1613 (45.8)
≥ detection limit	2172	33 (43.4)	33 (14.5)	236 (44.9)	1870 (53.1)
Missing	45	0 (0.0)	0 (0.0)	5 (1.0)	40 (1.1)

Values are presented as mean ± SD; all other variables are shown as number (%).

Abbreviations: MMI, methimazole; TRAb, thyrotropin receptor antibodies.

^*a*^The prediscontinuation MMI dose refers to the average daily maintenance dose before discontinuation, calculated based on the final 2 prescriptions.

The results of the multivariable regression analysis for the 4 MMI dose groups are presented in Table 2. In the crude model using the 2.5 mg/day group as the reference, the estimated RRs for relapse within 1 year were 0.95 (95% CI: 0.75-1.21) for the >1.25 to <2.5 mg/day group, 0.51 (95% CI: 0.32-0.83) for the 1.25 mg/day group, and 0.19 (95% CI: 0.05-0.75) for the <1.25 mg/day group. In the adjusted model, which accounted for age, sex, thyroid volume, smoking status, total duration of MMI treatment, duration of maintenance dose, and TRAb titer at the time of discontinuation, the associations remained consistent. Compared with the 2.5 mg/day group, the adjusted RRs were 0.92 (95% CI: 0.75-1.17) for the >1.25 to <2.5 mg/day group, 0.46 (95% CI: 0.28-0.75) for the 1.25 mg/day group, and 0.18 (95% CI: 0.05-0.73) for the <1.25 mg/day group. A statistically significant trend was observed across dose categories (*P* for trend < 0.05), suggesting a dose-response relationship where lower MMI doses prior to discontinuation were associated with a reduced risk of relapse.

**Table 2.** Association between prediscontinuation MMI dose and 1-year relapse of Graves’ disease: multivariable regression analysis

	Crude		Adjusted model	
Prediscontinuation MMI dose	RR	95% CI	RR	95% CI
<1.25 mg/day	**0.19**	**(0.05-0.75)**	**0.18**	**(0.05-0.73)**
1.25 mg/day	**0.51**	**(0.32-0.83)**	**0.46**	**(0.28-0.75)**
>1.25 to <2.5 mg/day	0.95	(0.75-1.21)	0.92	(0.73-1.17)
2.5 mg/day	Ref		Ref	

Bold values indicate statistically significant associations (*P* < .05).

Adjusted model: adjusted for age, sex, thyroid volume, smoking, duration of MMI treatment, duration of minimal MMI dose, thyrotropin receptor antibodies titer at the time of MMI discontinuation.

Abbreviations: CI, confidence interval; MMI, methimazole; RR, risk ratio.

In the propensity score-matched cohort (n = 172 in each group), covariate balance was adequately achieved, with standardized mean differences for all variables below 0.1 [Supplementary Table S1 (18)]. Patients receiving 1.25 mg/day of MMI had a lower 1-year relapse risk compared with those receiving 2.5 mg/day (RR: 0.44; 95% CI: 0.23-0.85; Table 3). The E-value for the observed RR was 3.97, and the E-value for the lower limit of the 95% CI was 1.63. In the propensity score-matched cohort comparing patients receiving ≤1.25 mg/day and those receiving >1.25 to ≤2.5 mg/day of MMI prior to discontinuation, each group included 271 patients (total n = 542). Covariate balance was achieved, with standardized mean differences for all variables below 0.1 [Supplementary Table S2 (18)]. The ≤1.25 mg/day group had a significantly lower 1-year relapse risk compared with the >1.25 to ≤2.5 mg/day group (RR: 0.46; 95% CI: 0.27-0.79; Table 4).

**Table 3.** Association between prediscontinuation MMI dose and 1-year relapse of Graves’ disease: propensity score-matched modified Poisson regression analysis

		RR	95% CI
**Prediscontinuation MMI dose**	**2.5 mg/day**	**Ref**	
	1.25 mg/day	**0.44**	**(0.23−0.85)**

Abbreviations: CI, confidence interval; MMI, methimazole; RR, risk ratio.

Bold values indicate statistically significant associations (*P* < .05).

**Table 4.** Association between prediscontinuation MMI dose (MMI ≤ 1.25 mg/day vs > 1.25 mg to ≤ 2.5 mg/day) and 1-year relapse of Graves’ disease: propensity score-matched modified Poisson regression analysis

		RR	95% CI
**Prediscontinuation MMI dose**	**> 1.25 mg to ≤2.5 mg/day**	**Ref**	
	≤ 1.25 mg/day	**0.46**	**(0.27-0.79)**

Abbreviations: CI, confidence interval; MMI, methimazole; RR, risk ratio.

Bold values indicate statistically significant associations (*P* < .05).

In a sensitivity analysis restricted to patients with at least 1 year of follow-up after MMI discontinuation, the results were consistent with those of the main and propensity score-matched analyses [RR: 0.48; 95% CI: 0.26-0.89 for 1.25 mg/day vs 2.5 mg/day (Supplementary Tables S3 and S4) (18); RR: 0.38; 95% CI: 0.22-0.68 for ≤1.25 mg/day vs >1.25 to ≤2.5 mg/day (Supplementary Tables S5 and S6) (18)]. In another sensitivity analysis excluding patients who received levothyroxine at the time of MMI discontinuation, the associations remained consistent with the primary findings: the adjusted RRs (95% CI) for relapse were 0.18 (0.04-0.73) for the <1.25 mg/day group, 0.43 (0.26-0.71) for the 1.25 mg/day group, and 0.89 (0.70-1.13) for the >1.25 to ≤2.5 mg/day group, using the 2.5 mg/day group as the reference.

## Discussion

Our study demonstrated that lower prediscontinuation doses of MMI were associated with a reduced risk of relapse in patients with Graves’ disease. This association was consistently observed in both propensity score-matched analyses comparing the 1.25 mg/day and 2.5 mg/day groups, as well as the ≤1.25 mg/day and >1.25 to ≤2.5 mg/day groups. These findings suggest that reducing the maintenance dose below 2.5 mg/day before discontinuation may help mitigate the risk of relapse.

To our knowledge, this is the first study to discuss the minimum maintenance dose of ATDs in the context of treatment strategies for Graves’ disease. While there is a consensus that ATDs should be tapered before discontinuation, previous studies have suggested that the duration of the maintenance period, rather than the minimum maintenance dose itself, may be associated with the risk of relapse. The minimum dose has received less attention in discussions, partly because it is often determined by the available tablet strengths. Previous studies have identified factors such as sex, age, treatment duration, and TRAb negativity as predictors of relapse. Our findings suggest that, in addition to these established factors, the minimum maintenance dose before discontinuation may be an independent determinant of relapse risk.

The mechanism by which tapering to a lower maintenance dose reduces the relapse rate remains unclear. Since treatment duration has been suggested to contribute to TRAb negativity, it is possible that lowering the minimum maintenance dose extended the overall treatment period, thereby reducing relapse rates. However, in our analysis, this association persisted even after adjusting for both treatment duration and the duration of maintenance at the minimum dose. This suggests that the minimum dose itself, as well as the gradual tapering process, may independently reduce the risk of relapse. One potential explanation is that gradual tapering to a lower maintenance dose, such as ≤1.25 mg, may help reduce the risk of relapse by stabilizing the immune state or minimizing immune reactivation. However, these remain speculative hypotheses, and further research is needed to elucidate the underlying immunological and physiological mechanisms of these observations.

In clinical practice, the minimum maintenance dose is a modifiable factor, and clinicians should actively adjust it based on patient characteristics to minimize the risk of relapse. While lower doses were associated with longer treatment durations, most adverse events related to ATDs typically occur within the first 3 months of treatment and tend to decrease with lower doses (5, 11, 26). From a healthcare policy perspective, promoting the development and distribution of lower-dose formulations, such as 2.5 mg and 1.25 mg tablets, could enable more precise dosing strategies. Compared to developing new drugs, ensuring the availability of lower-dose formulations is a cost-effective approach to improving the safety and efficacy of existing treatments. If further research confirms this association, these findings could contribute to revisions in clinical guidelines and encourage the broader adoption of tailored tapering strategies in clinical practice. These strategies could substantially reduce the number of relapsed cases, considering the global prevalence of Graves’ disease.

This study has several limitations that should be considered when interpreting the results.　First, misclassification of relapse is a potential concern. We defined relapse as resuming ATD therapy rather than using the American Thyroid Association's remission criteria. Thus, cases of mild subclinical hyperthyroidism managed without immediate treatment may have been misclassified as nonrelapse, potentially underestimating the true relapse rate. However, because the same definition was applied uniformly across groups, any misclassification was likely nondifferential and may have biased the results toward the null, making the observed effect conservative (27). Second, as this was an observational study, residual confounding due to unmeasured variables cannot be entirely ruled out. For example, information on the presence or severity of Graves’ orbitopathy and the use of immunosuppressive therapy was not systematically available and thus could not be included in the analysis. However, in Japan, immunosuppressive therapy for Graves’ orbitopathy typically refers to systemic steroid use, and such therapy has not generally been performed at our institution. This may further reduce the likelihood of confounding related to this factor. In addition, the E-value for the observed association (3.97) shows that an unmeasured confounder would need to be strongly associated with both the exposure and outcome. Even for the lower limit of the 95% CI (E-value 1.63), a moderately strong confounder would still be required to nullify the association. Third, patients who successfully maintained euthyroidism on 1.25 mg/day may have inherently lower relapse risk, raising the possibility of survivor bias. Although we adjusted for several variables known to be associated with relapse risk, this structural bias cannot be fully eliminated, and the observed risk difference may have been partially overestimated. Fourth, this study was conducted at a specialized thyroid center, which may affect its generalizability. However, the demographic characteristics of our study population (mean age 45.3 ± 14.4 years; 81.8% female) are comparable to those reported in international epidemiological studies (28), although large-scale epidemiological data on Graves’ disease in Japan are limited. Fifth, some patients may have initially been diagnosed with Graves’ disease at another hospital years earlier and subsequently experienced a relapse, introducing heterogeneity into the study population. Future studies should distinguish between first-onset and relapsed cases to clarify their respective risks. Sixth, the short availability of 2.5 mg tablets limited our analysis to relapse within 1 year after MMI discontinuation. The long-term impact of lower maintenance doses remains unknown and warrants further study. Seventh, the limited sample size prevented us from evaluating potential effect modification in the association between lower maintenance doses and relapse risk. Relapse risk may vary by patient characteristics such as psychosocial stress and related socioeconomic status, which deserve further exploration. Finally, as this was a retrospective observational study, prospective studies with a prespecified design are warranted to validate and extend our findings. Additionally, the transportability of our findings outside Japan requires caution, as genetic differences (29, 30) and iodine intake variations (31) may influence relapse risk. Further studies are needed to assess their applicability across diverse populations.

In conclusion, our study suggests that lower minimum maintenance doses of MMI before discontinuation may be associated with a reduced risk of relapse in patients with Graves’ disease. Since the minimum maintenance dose is a modifiable factor in clinical practice, physicians should recognize the potential importance of dose reduction in selected patients.

## Data Availability

Data are available upon reasonable request.
