# Correction to: “Impact of Minimal Dose Strategy Before Antithyroid Drug Discontinuation on Relapse Risk in Graves’ Disease”

**DOI:** 10.1210/clinem/dgag016

**Published:** 2026-01-22

In the above-mentioned article by Miyamura K, Ito M, Yamaoka H, Hisakado M, Nishihara E, Fukata S, Nishikawa M, Miyauchi A, and Akamizu T (*J Clin Endo Metab*. 2025; doi: 10.1210/clinem/dgaf433), there were errors in the in-text reference citations.

In the originally published article, in the Materials and Methods section, the second sentence of the Relapse of Graves’ Disease subsection read “While the American Thyroid Association guideline defines remission as maintaining normal thyroid function for 1 year after discontinuation (15), cases of transient thyrotoxicosis following withdrawal of ATDs have also been reported (16).” The first reference citation has been changed from “15” to “8.” The sentence now reads “While the American Thyroid Association guideline defines remission as maintaining normal thyroid function for 1 year after discontinuation (8), cases of transient thyrotoxicosis following withdrawal of ATDs have also been reported (16).”

In the originally published article, in the Materials and Methods section, the ninth sentence of the Covariates subsection read “Since longer treatment duration has been associated with an increased cumulative remission rate (23), treatment duration was treated as a continuous variable.” The reference citation has been changed from “23” to “6.” The sentence now reads “Since longer treatment duration has been associated with an increased cumulative remission rate (6), treatment duration was treated as a continuous variable.”

In the originally published article, in the Materials and Methods section, the tenth sentence of the Covariates subsection read “TRAb titers at the time of MMI discontinuation were categorized as < detection limit or ≥ detection limit, as nonnegative TRAb levels have been associated with a higher risk of relapse (24).” The reference citation has been changed from “24” to “9.” The sentence now reads “TRAb titers at the time of MMI discontinuation were categorized as < detection limit or ≥ detection limit, as nonnegative TRAb levels have been associated with a higher risk of relapse (9).”

In the originally published article, in the Materials and Methods section, the fifth sentence of the Statistical Analyses subsection read “Since the risk of relapse was 13.1%, we used modified Poisson regression with robust variance estimation to calculate risk ratios (RRs) for each dose category (25).” The reference citation has been changed from “25” to “24.” The sentence now reads “Since the risk of relapse was 13.1%, we used modified Poisson regression with robust variance estimation to calculate risk ratios (RRs) for each dose category (24).”

In the originally published article, in the Materials and Methods section, the sixth sentence of the Sensitivity Analysis subsection read “The E-value was computed based on the RR and its CI limits (26).” The reference citation has been changed from “26” to “25.” The sentence now reads “The E-value was computed based on the RR and its CI limits (25).”

The article has been corrected online.

doi: 10.1210/clinem/dgaf433

